# Case−Control Study of Risk Factors for Meningococcal Disease in Chile

**DOI:** 10.3201/eid2307.160129

**Published:** 2017-07

**Authors:** Andrea Olea, Isabel Matute, Claudia González, Iris Delgado, Lucy Poffald, Elena Pedroni, Tania Alfaro, Macarena Hirmas, Manuel Nájera, Ana Gormaz, Darío López, Sergio Loayza, Catterina Ferreccio, Doris Gallegos, Rodrigo Fuentes, Pablo Vial, Ximena Aguilera

**Affiliations:** Universidad del Desarrollo, Santiago, Chile (A. Olea, I. Matute, C. González, I. Delgado, L. Poffald, M. Hirmas, M. Nájera, A. Gormaz, R. Fuentes, P. Vial, X. Aguilera);; Universidad Isalud, Buenos Aires, Argentina (E. Pedroni);; Universidad de Chile, Santiago (T. Alfaro);; Chilean Ministry of Health, Santiago (D. López, S. Loayza, D. Gallegos);; Pontificia Universidad Católica de Chile, Santiago (C. Ferreccio)

**Keywords:** meningococcal disease, meningitis/encephalitis, serogroups, risk factors, case−control study, Neisseria meningitidis, bacteria, epidemiology, Chile

## Abstract

An outbreak of meningococcal disease with a case-fatality rate of 30% and caused by predominantly serogroup W of *Neisseria meningitidis* began in Chile in 2012. This outbreak required a case−control study to assess determinants and risk factors for infection. We identified confirmed cases during January 2012−March 2013 and selected controls by random sampling of the population, matched for age and sex, resulting in 135 case-patients and 618 controls. Sociodemographic variables, habits, and previous illnesses were studied. Analyses yielded adjusted odds ratios as estimators of the probability of disease development. Results indicated that conditions of social vulnerability, such as low income and overcrowding, as well as familial history of this disease and clinical histories, especially chronic diseases and hospitalization for respiratory conditions, increased the probability of illness. Findings should contribute to direction of intersectoral public policies toward a highly vulnerable social group to enable them to improve their living conditions and health.

Meningococcal disease has a case-fatality rate of 50% for patients not given treatment and 10%–20% for those given treatment (*1**,**2*). The causative agent is *Neisseria meningitidis*, for which 14 serogroups have been identified; 6 (A, B, C, W, X, and Y) can cause human disease (*3*). Geographic distribution and epidemic potential differ for each serogroup (*3*). Serogroups A, B, and C are responsible for 80%–90% of cases worldwide, and serogroups Y and W account for the remaining 10%–20%. The extended meningitis belt of sub-Saharan Africa has the highest frequencies of this disease. Before 2010 and mass preventive vaccination campaigns during the MenAfriVac project (http://www.meningvax.org/), group A meningococcus accounted for ≈80%–85% of all cases in the meningitis belt; epidemics occur at intervals of 7–14 years. Since that time, the frequency of serogroup A has decreased, including carriage (*4*), but other meningococcal serogroups, such as W, X, and C, still cause epidemics, albeit at a lower frequency and smaller size (*2*).

The principal clinical forms of meningococcal disease are meningeal syndrome, meningococcal sepsis, and pneumonia. Mortality rates increase for meningococcal sepsis (8%–13%), and rates for sepsis with shock can reach 34%–73% (*5*). Sequelae are present in 10%–20% of patients; the most common ones are limb necrosis, neurologic impairment, and deafness (*1*). Risk factors for disease development and death include individual characteristics, environmental and living conditions, and access to healthcare. Socially disadvantaged persons are at greater risk for disease development and have less access to healthcare resources; thus, illness and death rates for these persons are higher than for those in more privileged social positions (*6**,**7*).

In the 1990s in Chile, the incidence rate of meningococcal disease was stable (≈3.5 cases/100,000 persons). This rate began to decrease gradually in 2001 and eventually reached 0.5 cases/100,000 persons by 2010 (a low level of endemicity) (*5*). The predominant serogroup until 2012 was serogroup B. During this time, there were also small outbreaks of serogroup C disease that were controlled with vaccines and isolated cases of serogroup W disease, which made up only 2% of the total. However, the incidence of meningococcal disease began to increase in 2010 because of an increase in serogroup W (*5*), which by 2012 represented 58% of all cases (Figure). This increase in serogroup W occurred simultaneously with an increased mortality rate (from 10% to 30%) and nonspecific clinical symptoms and sequelae such as amputations, hearing loss, and neurologic damage in 10% of case-patients (*8*). As a result of this increase in meningococcal disease, a vaccination campaign was implemented in Chile at the end of 2012 with a quadrivalent meningococcal conjugate vaccine for serotypes A, C, W, and Y for children <5 years of age.

**Figure:**
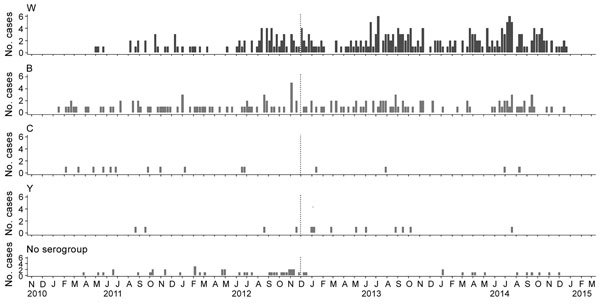
Monthly cases of meningococcal disease by serogroup, Chile 2011–2014. Dotted vertical line indicates beginning of the vaccination campaign against *Neisseria meningitidis* serogroups A, C, W, and Y. Data were obtained from the Department of Epidemiology, Ministry of Health of Chile.

In this context, the Ministry of Health of Chile considered it necessary to conduct a study to identify factors associated with development of meningococcal disease. This study was based on the hypothesis that occurrence of this disease is related to living conditions, lifestyles, and medical histories of infected persons.

## Methods

We conducted a population-based, case−control study to identify risk factors for meningococcal disease. The study included regions in Chile where >3 cases of disease occurred during January 2012−March 2013. All confirmed cases of meningococcal disease during this period were included; there were no age or nationality restrictions, including deceased patients. Cases were identified by using the monitoring system of the Ministry of Health of Chile because regulations in Chile require that all cases in this country must be reported immediately (*9*). Cases were confirmed by isolating *N. meningitidis* from cerebrospinal fluid cultures or cultures of other normally sterile fluids or tissues, or by PCR for cerebrospinal fluid or plasma (*1*). All strains were confirmed and serotyped at Public Health Institute of Chile by the slide agglutination test (*10*).

We assigned 4 controls per case-patient, which enabled 98% power to detect 50% more overcrowding in case-patients than in controls, a variable that is a risk factor for meningococcal disease (*11**–**14*) and is reliable and available for Chile. Controls lived in the same municipalities as case-patients, resided in Chile during the study period, and had not been given a diagnosis of meningococcal disease or meningeal syndrome of unknown etiology. Group matching for sex and age was performed by using a proportional distribution of controls corresponding to the number of inhabitants in each municipality and the proportions of sex and age (<2, 2–6, 7–18, 19–45, 46–65, and >66 years) observed for case-patients. Controls were selected by using a 3-stage random sampling procedure (blocks, homes, and persons) based on the most recent census data available. Controls who refused to participate were replaced in the same area by means of a systematic skipping of dwellings.

We studied case-patients and controls by using a structured questionnaire that contained 118 questions, including variables related to social and environmental determinants (education, occupation, income, healthcare system, housing, overcrowding); individual factors (sex, age, ethnicity, habits such as alcohol and tobacco use, and medical history); and vaccination history for meningococcal disease (vaccine against serogroups A, B, and C and quadrivalent meningococcal conjugate vaccine against serogroups A, C, W, and Y). Vaccination status and date were verified by vaccination card for children <5 years of age and through self-report for persons >5 years of age. We explicitly asked case-patients about the period before their disease. We asked controls about their living conditions and lifestyles at the time of the survey and <2 years before to coincide with exposure periods of case-patients. For persons <14 years of age or those who died, the survey was conducted with a relative. Collection of information was conducted by physicians, public health professionals, and epidemiologists during August 16−November 22, 2013.

We captured information by using the SurveyToGo program (http://www.dooblo.net/stgi/downloads.aspx) and exported the database into SPSS version 21.0 (IBM, Armonk, NY, USA), which was validated data on a weekly basis. Descriptive and bivariate analyses were conducted. Measures of central tendency and dispersion were calculated for quantitative variables, and frequencies and percentages were used for categorical variables. Statistical tests were used to identify significant differences (p<0.05 in a 1-sided test) between the case-patients and controls. A Fisher exact or χ^2^ test was used for categorical variables, and a Mann-Whitney test was used for continuous variables, after checking that these variables did not distribute normally by the Shapiro–Wilk test. Further analyses of significant results involved calculating raw odds ratios (ORs) and 95% CIs.

The effect of exposure variables was evaluated by using multivariate logistic regression models to estimate the probability of development of meningococcal disease, which produced adjusted ORs and 95% CIs. Multivariate models included variables that were significant in bivariate analysis, which was conducted by using the forward stepwise method. In each model, the goodness of fit (Nagelkerke R^2^ value) and significance were evaluated. Matching enabled controlling by sex, age, and region of residence. Three models were used: 1 for children <5 years of age, 1 for persons >5 years of age, and 1 that considered only serogroup W disease for all ages in Santiago (Metropolitan Region). Serogroup W was not analyzed by age groups because of low numbers of cases.

The protocol and consent and assent forms were approved by the Ethics Committee of Faculty of Medicine, Clínica Alemana Universidad del Desarrollo (Santiago, Chile). All participants were informed of the objectives of the study and voluntarily agreed to participate anonymously and with their confidentiality protected by signing a consent form. Contact information for the cases was obtained from records of the Ministry of Health of Chile in compliance with current regulations (*11**–**13*). A psychological support strategy was also developed for relatives of deceased case-patients and those with serious sequelae, which included the presence of a psychologist during the interview and a referral to the corresponding health network when requested.

## Results

Of 149 case-patients reported in regions with >3 cases, where ≈82% of the population resided, 135 (90.6%) were enrolled in the study; 8 case-patients could not be located, and 6 case-patients refused to participate. A total of 618 controls were enrolled in the study.

More than half (55%) of case-patients and controls were men. There were no age differences between case-patients and controls, except for infants <1 year of age, who had a higher proportion of cases (cases 29%, controls 13%). Of all case-patients and controls, 60% lived in the metropolitan region, 98% had Chilean nationality, and <9% belonged to the native population. A total of 46.6% of case-patients had disease caused by serogroup W, 31.8% by serogroup B, 2.5% by serogroup C, and 0.7% by serogroup Y; 18.2% of cases were not serogrouped. For outcomes, 27% of case-patients died <43 days after the first consultation (median 1 day), all during hospitalization for meningococcal disease; 14.2% survived and had sequelae (neurologic, 8 case-patients; amputations, 4; sensory, 3); and 58.8% survived without any short-term sequelae.

Because meningococcal disease differs in frequency by patient age and serogroups, we analyzed persons <5 years of age and those >5 years of age. We also analyzed case-patients who had serogroup W in the Metropolitan Region, which contained most cases with this serogroup.

### Analysis for Persons <5 Years of Age

We analyzed 59 case-patients and 281 controls <5 years of age; of these persons <5 years of age, 66% of case-patients and 28% of controls were <1 year of age. Bivariate analysis showed a strong association between meningococcal disease and social and environmental characteristics, such as overcrowding (>2.5 persons/bedroom), fewer years of mother’s education, belonging to the public health insurance system, and monthly household income <US $517, which corresponded to the threshold of the lowest quintile income in the country in 2011 (*14*). Among habits, greeting >2 persons with a kiss on the mouth was associated with development of disease. For medical history of children, previous hospitalizations for asthma or acute lower respiratory tract infection (RTI), pertussis (whooping cough), and diarrhea were risk factors. A family history of meningococcal disease also increased risk for illness (Table 1).

**Table 1:** Meningococcal disease determinants for persons <5 years age and controls for all *Neisseria meningitidis* serogroups, Chile, January 2012−March 2013*

Characteristic	Case-patients, n = 59	Controls, n = 281	p value	OR (95% CI)
Demographic				
Age, y†	0 (0–1)	1 (0–2)	<0.001‡	NA
Age <1 y†	39/59	80/281	<0.001§	4.9 (2.7–8.9)
Male sex	42/59	187/281	0.298§	1.2 (0.7–2.3)
Chilean nationality	58/59	278/281	0.535§	0.6 (0.1–6.1)
Native person	7/59	22/281	0.220§	1.6 (0.6–3.9)
Residence in Santiago (metropolitan region)	37/59	179/281	0.499§	1.0 (0.5–1.7)
Social and environmental				
No. bedrooms in home†	3 (2–3)	3 (2–4)	0.045‡	NA
No. persons/bedroom†	2 (1.7–3.0)	1.8 (1.3–2.1)	<0.001‡	NA
Square feet of housing	646 (484–1,023)	753 (517–1,076)¶	0.323‡	NA
Square feet/person	129 (97–180)	143 (93–230)¶	0.212‡	NA
Education for mother, y†	13 (111–13)	13 (12–15)	0.015‡	NA
Children not attending school in mother’s care†	39/47	144/214	0.022§	1.4 (1.1–5.3)
Lived in crowded places†	3/59	0/281	0.005§	NA
Income <US $517†	34/59	101/262	0.006§	2.2 (1.2–3.8)
Public health insurance†	64/59	209/281	0.003§	2.2 (1.0–4.8)
Shared bedroom with >2 persons†	51/59	180/281	<0.001§	3.6 (1.6–7.8)
Shared bed with >2 persons†	24/59	59/281	0.002§	2.6 (1.4–4.7)
Shared space with other children†	54/59	212/281	0.003§	3.5 (1.4–9.1)
Overcrowding†	22/59	55/281	0.004§	2.4 (1.3–4.5)
Individual behavior and medical history				
No. hospitalizations†	0 (0–1)	0 (0–1)	0.004‡	NA
No. nonrespiratory chronic diseases	0 (0–0)	0 (0–0)	0.093‡	NA
Acute respiratory illness	33/59	186/281	0.090§	1.5 (0.9–2.7)
Bronchopneumonia†	8/59	7/281	0.001§	6.1 (2.1–17.7)
Greeted >2 persons with kiss on mouth†	24/59	70/281	0.012§	2.1 (1.2–3.7)
History of meningococcal disease in family†	13/59	24/281	0.004§	3.0 (1.4–6.4)
History of hospitalizations†	28/59	84/281	0.008§	2.1 (1.2–3.8)
Hospitalization for asthma or acute lower RTI†	19/59	44/281	0.004§	2.6 (1.4–4.8)
Hospitalization for whooping cough†	4/59	1/281	0.004§	20.4 (2.2–185.7)
Hospitalization for diarrhea†	4/59	4/281	0.033§	5.0 (1.2–20.7)
Nonrespiratory chronic disease	12/59	35/281	0.086§	1.8 (0.9–3.7)
Stressful event	14/59	42/281	0.076§	1.8 (0.9–3.5)

*Values are median (IQR) or no. positive/no. tested except as indicated. IQR, interquartile range; NA, not applicable; OR, odds ratio; RTI, respiratory tract infection. †Significant difference. ‡By Mann-Whitney test. §By Fisher exact test. ¶n = 276.

Because the vaccination campaign began at the end of 2012, most case-patients had no access to the vaccine before the disease developed. A total of 70.6% of controls <5 years of age had received the vaccine, in contrast to only 1 (1.7%) child among case-patients. However, serogroup B was not included in the vaccine. There is strong evidence that vaccination with quadrivalent meningococcal conjugate vaccine is protective (*4**,**15**–**18*). Because case-patients did not have the same probability of being vaccinated as controls, vaccination status was not considered in the analysis.

The multivariate model included variables significant in the bivariate model. The multivariate model showed that being <1 year of age, having a monthly household income <US $517, sharing daily activities with other children, sharing the same bed with >2 persons, greeting >2 persons with a kiss on the mouth, hospitalization for whooping cough and asthma or acute lower RTI, and a familial history of meningococcal disease were primary determinants for this disease in children <5 years of age (Table 2).

**Table 2:** Multivariate model of risk factors for development of meningococcal disease in persons <5 years of age for all *Neisseria meningitidis* serogroups, Chile, January 2012−March 2013*

Risk factor	β	SE	p value	OR (95% CI)
Hospitalization for whooping cough	2.94	1.30	0.024	18.86 (1.47–241.87)
Age <1 y	2.04	0.38	<0.001	7.68 (3.68–16.05)
Shared space with other children	1.78	0.63	0.005	5.91 (1.73–20.14)
Hospitalization for asthma or acute lower RTI	1.44	0.41	<0.001	4.24 (1.91–9.44)
History of meningococcal disease in family	1.39	0.46	0.002	4.02 (1.63–9.92)
Shared bed with >2 persons	1.22	0.38	0.001	3.37 (1.62–7.03)
Income <US $517	0.95	0.35	0.007	2.59 (1.30–5.17)
Greeted >2 persons with kiss on mouth	0.85	0.37	0.023	2.35 (1.13–4.89)
Constant	–6.87	1.56	<0.001	0.00

*OR, odds ratio; RTI, respiratory tract infection; R^2^ = 39%.

### Analysis for Persons >5 Years of Age

We analyzed 76 case-patients and 337 controls >5 years of age. Bivariate analysis showed a strong association between the probability of development of meningococcal disease and overcrowding (fewer bedrooms, fewer overall square feet, and fewer square feet per person). Meningococcal disease was also strongly associated with having lived in crowded places, such as regiments, hospitals, or campgrounds; having had a stressful event (death of close person, moving, divorce, or losing a job); and excessive use of alcohol in persons >14 years of age (>4 or more glasses of alcohol at each drinking opportunity or until drunk). For medical history, having had an episode of acute respiratory illness, having been hospitalized for asthma or acute lower RTI, and having >1 nonrespiratory chronic illness increased the probability of development of meningococcal disease. For use of medications, an association was found between disease and regular use of corticoids and antidepressants (Table 3).

**Table 3:** Meningococcal disease determinants for persons >5 years of age and controls for all *Neisseria meningitidis* serogroups, Chile, January 2012−March 2013*

Characteristic	Case-patients, n = 76	Controls, n = 337	p value	OR (95% CI)
Demographic				
Age, y	35 (6–57)	36 (18–55)	0.849†	NA
Age <25 y	26/76	115/337	0.544‡	1.0 (0.6–1.7)
Male sex	33/76	153/337	0.427‡	0.9 (0.6–1.5)
Chilean nationality	75/76	329/337	0.484‡	1.8 (0.2–14.8)
Native person	5/76	30/337	0.347‡	0.7 (0.3–1.9)
Residence in Santiago (metropolitan region)	44/76	190/337	0.457‡	1.1 (0.6–1.8)
Social and environmental				
No. bedrooms in home§	3 (2–4)	4 (2-4)	0.042†	NA
No. persons/bedroom	1.3 (1–2)	1.3 (1–1.7)	0.146†	NA
Square feet of housing§	753 (505–1,103)¶	861 (581–1,173)#	0.038†	NA
Square feet/person§	179 (108–288)¶	215 (143–375)#	0.031†	NA
Education for mother of persons <18 y of age, y	13 (9–14)**	13 (13–16)††	0.063†	NA
Lived in crowded places§	5/76	7/337	0.050‡	3.3 (1.0–10.8)
Income <US $517	33/74	126/317	0.263‡	1.2 (0.7–2.0)
Public health insurance§	64/76	246/337	0.026‡	2.0 (1.0–3.8)
Shared bedroom with >2 persons	20/76	71/337	0.198‡	1.3 (0.8–2.4)
Shared bed with >2 persons	3/76	18/337	0.439‡	0.7 (0.2–2.5)
Overcrowding§	12/76	24/337	0.018‡	2.4 (1.2–5.1)
Individual behavior and medical history				
No. hospitalizations	1 (0–2)	1 (0–2)	0.707†	NA
No. nonrespiratory chronic diseases§	1 (0–2)	1 (0–1)	0.005†	NA
Acute respiratory illness§	42/76	141/337	0.023‡	1.7 (1.0–2.8)
Bronchopneumonia	3/76	6/337	0.219‡	2.3 (0.6–9.3)
Person >14 y of age consuming >4 alcoholic drinks§	14/59	35/269	0.034‡	2.1 (1.0–4.2)
Depression§	16/76	43/337	0.050‡	1.8 (1.0–3.4)
History of meningococcal disease in family	11/76	42/337	0.378‡	1.2 (0.6–2.4)
History of hospitalizations	46/76	200/337	0.478‡	1.1 (0.6–1.7)
Hospitalization for asthma or acute lower RTI§	12/76	26/337	0.029‡	2.2 (1.1–4.7)
Hospitalization for diarrhea	3/76	10/337	0.439‡	1.3 (0.4–5.0)
Hypertension§	14/76	34/337	0.037‡	2.0 (1.0–4.0)
Nonrespiratory chronic disease§	49/76	158/337	0.004‡	2.1 (1.2–3.4)
Stressful event§	49/76	179/337	0.047‡	1.6 (1.0–2.7)
Used corticoids§	8/76	11/337	0.012‡	3.5 (1.4–9.0)
Used antidepressants§	10/76	22/337	0.049‡	2.2 (1.0–4.8)

*Values are median (IQR) or no. positive/no. tested except as indicated. IQR, interquartile range; NA, not applicable; OR, odds ratio; RTI, respiratory tract infection. †By Mann-Whitney test. ‡By Fisher exact test. §Significant difference. ¶n = 74. #n = 333. **n = 22. ††n = 82.

We also performed multivariate analysis for this group. Multivariate analysis showed that living lived in overcrowded household or in collective places, excessive use of alcohol for persons >14 years of age, regular use of corticoids, having >1 nonrespiratory chronic illness, and having been hospitalized for asthma or acute lower RTI were related to development of disease (Table 4).

**Table 4:** Multivariate model of risk factors for development of meningococcal disease in persons >5 years of age for all *Neisseria meningitidis* serogroups, Chile, January 2012−March 2013*

Risk factor	β	SE	p value	OR (95% CI)
Overcrowding	1.61	0.50	0.001	5.01 (1.87–13.45)
Lived in crowded places	1.51	0.66	0.022	4.54 (1.24–16.67)
Used corticoids	1.46	0.57	0.011	4.29 (1.39–13.21)
Person 14 y of age consuming >4 alcoholic drinks	1.30	0.41	0.001	3.68 (1.66–8.19)
Nonrespiratory chronic disease	1.20	0.36	0.001	3.32 (1.64–6.73)
Hospitalization for asthma or acute lower RTI	1.08	0.49	0.028	2.94 (1.12–7.71)
Constant	−6.73	1.69	<0.001	0.00
*OR, odds ratio; RTI, respiratory tract infection; R^2^ = 19%.				

### Analysis for Serogroup W in Metropolitan Region of Chile

For case-patients with meningococcal disease caused by serogroup W, 82% occurred in the metropolitan region of Santiago. This region had a greater risk for disease caused by serogroup W than other regions, where serogroup B predominated. Thus, we analyzed cases caused by serogroup W in the metropolitan region (50 case-patients and 366 controls).

The most affected group was children <1 year of age (28% for cases; 15% for controls). Meningococcal disease was strongly associated with overcrowding and conditions that increased proximity between persons, such as sharing the same bedroom and sharing daily activities with other children <5 years of age. For medical history, risk for meningococcal disease was increased in persons who had previous hospitalizations, especially for respiratory diseases. A history of chronic diseases (diabetes, obesity, depression, and hypertension) and use of medications also increased the risk for meningococcal disease (Table 5).

**Table 5:** Meningococcal disease determinants for case-patients and controls for *Neisseria meningitidis* serogroup W, Santiago (Metropolitan Region) of Chile, January 2012−March 2013*

Characteristic	Case-patients, n = 50	Controls, n = 366	p value	OR (95% CI)
Demographic				
Age, y	3.5 (0–51.5)	5 (1–38)	0.765†	NA
Age <1 y‡	14/50	56/366	0.005§	2.6 (1.3–5.0)
Age <5 y	26/50	179/366	0.398§	1.1 (0.6–2.4)
Male sex	28/50	203/366	0.534§	1.0 (0.6–1.9)
Chilean nationality	50/50	357/366	0.312§	NA
Native person	9/50	37/366	0.082§	2.0 (0.9–4.3)
Social and environmental				
No. bedrooms in home	3 (2–4)	3 (2–4)	0.273†	NA
No. persons/bedroom	1.7 (1.2–2.5)	1.5 (1.2–2.0)	0.135†	NA
Square feet of housing	614 (503–1,076)	823 (546–1,206)	0.053†	NA
Square feet/person	157 (93–273)	189 (120–301)	0.146†	NA
Education for mother of persons <18 y of age, y	13 (11–15)¶	13 (13–15)#	0.274†	NA
Children <5 y of age not attending school in mother’s care	13/19	89/137	0.492§	1.2 (0.4–3.3)
Lived in crowded places	0/50	3/366	0.680§	NA
Income <US $517	22/50	111/366	0.073§	1.6 (0.9–3.0)
Public health insurance‡	42/50	259/366	0.032§	2.2 (1.0–4.8)
Shared bedroom with >2 persons‡	26/50	137/366	0.035§	1.8 (1.0–3.3)
Shared bed with >2 persons	8/50	45/366	0.294§	1.4 (0.6–3.1)
Children <5 y of age sharing space with other children‡	25/26	134/179	0.008§	8.4 (1.1–63.7)
Overcrowding‡	14/50	48/366	0.008§	2.6 (1.3–5.1)
Individual behavior and medical history				
No. hospitalizations	1 (0–2)	0 (0–1)	<0.001†	NA
No. nonrespiratory chronic diseases	1 (0–2)	0 (0–1)	0.002†	NA
Acute respiratory illness	32/50	204/366	0.170§	1.4 (0.8–2.6)
Bronchopneumonia	4/50	11/366	0.093§	2.8 (0.9–9.2)
Person >14 y of age consuming >4 alcoholic drinks	5/22	22/148	0.246§	1.7 (0.6–5.0)
Children <5 y of age greeting >2 persons with kiss on mouth	11/26	45/179	0.058§	2.2 (0.9–5.1)
Depression‡	9/50	26/366	0.015§	2.9 (1.3–6.5)
Diabetes mellitus ‡	6/50	14/366	0.023§	3.4 (1.3–9.4)
History of meningococcal disease in family	8/50	43/366	0.256§	1.4 (0.6–3.2)
History of hospitalizations‡	33/50	162/366	0.003§	2.4 (1.3–4.5)
Hospitalization for asthma or acute lower RTI‡	15/50	42/366	0.001§	3.3 (1.7–6.6)
Hospitalization for whooping cough‡	3/50	0/366	0.002§	NA
Hospitalization for diarrhea	2/50	6/366	0.248§	2.5 (0.5–12.7)
Hypertension‡	7/50	19/366	0.026§	3.0 (1.2–7.5)
Nonrespiratory chronic disease‡	30/50	112/366	<0.001§	3.4 (1.9–6.2)
Obesity‡	7/50	21/366	0.038§	2.7 (1.1–6.6)
Stressful event	23/50	126/366	0.076§	1.6 (0.9–2.9)
Used medicines‡	22/50	101/366	0.015§	2.1 (1.1–3.8)
Used corticoids	4/50	18/366	0.265§	1.7 (0.5–5.2)
Used antidepressant drugs‡	5/50	8/366	0.013§	5.0 (1.6–15.9)
Used antihypertension drugs‡	10/50	25/366	0.005§	3.4 (1.5–7.6)
Used hypoglycemic agents‡	6/50	17/366	0.045§	2.8 (1.0–7.5)

*Values are median (IQR) no. or no. positive/no. tested. IQR, interquartile range; NA, not applicable; OR, odds ratio; RTI, respiratory tract infection. †By Mann-Whitney test. ‡Significant results. §By Fisher exact test. ¶n = 29. #n = 224.

Multivariate analysis showed that the demographic and social factors that remained associated with development of disease were age <1 year (≈4 times higher) and overcrowding. For clinical history, the probability of development of meningococcal disease was 5.8 times higher for those who had had >1 nonrespiratory chronic illness and ≈3 times higher for those previously hospitalized for asthma or acute lower RTI (Table 6).

**Table 6:** Multivariate model of risk factors for development of meningococcal disease caused by *Neisseria meningitidis* serogroup W, Santiago (metropolitan region) of Chile, January 2012−March 2013*

Risk factor	β	SE	p value	OR (95% CI)
Nonrespiratory chronic disease	1.76	0.37	<0.001	5.78 (2.78–12.04)
Age <1 y	1.44	0.42	0.001	4.23 (1.86–9.60)
Hospitalization for asthma or acute lower RTI	1.04	0.38	0.006	2.83 (1.35–5.95)
Overcrowding	0.84	0.40	0.034	2.32 (1.07–5.06)
Constant	−4.38	1.04	<0.001	0.01

*OR, odds ratio; RTI, respiratory tract infection; R^2^ = 20%.

## Discussion

We sought to understand the principal determinants and risk factors for development of meningococcal disease in Chile and included 91% of the cases of this disease in Chile in 2012 and the first 3 months of 2013. Our study identified some risk factors previously reported and some that were not previously reported. These factors were a combination of social conditions, habits, and host health status, which are fundamental for the infectious process.

Evidence indicates that living conditions are one of the key factors in the likelihood of development of meningococcal disease (*19**–**22*). This evidence was especially true when linked with a lower socioeconomic level, as was the finding in our study for beneficiaries of the public health system, those who had monthly incomes <US $517, and those children whose mothers had less education.

For housing, 1 study showed that the number of persons per bedroom (>1.5) or those sharing the room or the bed were risk factors for meningococcal disease (*23*). In our study, we found that the average number of bedrooms was smaller and that overcrowding was greater for case-patients than for controls. Controls had an average of 41 more square feet per person in their houses than case-patients. We also found a higher probability of development of meningococcal disease in persons <5 years of age who shared a bedroom or bed with >2 persons.

For habits of persons, excessive use of alcohol in case-patients >14 years of age was twice as common as in controls, which is consistent with results of other studies (*19**,**24**,**25*). For exposure to tobacco, in contrast with findings of other studies (*19**,**21**,**26**–**30*), we found no association in our study.

Another major risk factor was proximity to other persons, in which a higher amount of contacts is associated with a greater likelihood of development of meningococcal disease (*19**,**22**,**25**–**27**,**31**–**36*). Our study showed that having contact with other children during the day and greeting >2 persons with a kiss on the mouth were risks for development of disease in preschoolers. Persons >5 years of age who had lived in crowded places also had a greater likelihood for development of meningococcal disease than persons who had not lived in such places.

For previous clinical history, other studies reported that patients with serious medical problems have a greater risk for development of meningococcal disease, especially patients with nephrotic syndrome, systemic lupus erythematosus and liver disease (*19**,**26*), HIV or other immunosuppressive diseases (*25**,**26*), a history of corticoid use (*26*), decreased endothelial thrombomodulin expression (*19**,**26**,**35**–**37*), and anatomic or functional asplenia (*19**,**25**,**35*). Stress and depression (*34*) have also been shown to be risk factors, and low weight or obesity (*26*) might predispose a person to development of meningococcal disease.

We found a greater probability of development of meningococcal disease in persons with a history of previous hospitalization for asthma or acute lower RTI in both age groups studied. In persons >5 years of age, we showed that those who have a chronic disease and use more medications, especially corticoids, on a regular basis had a higher probability of development of meningococcal disease. For patients >5 years of age who had chronic diseases, hypertension was present in 18.4% of case-patients but only 10.1% of controls. This finding might be the result of persons who had meningococcal disease and more previous diseases or hospitalizations; thus their history of hypertension was better known.

Family history has been reported as a risk factor for meningococcal disease (*21**,**35**–**38*), which indicates that genetic polymorphisms might be associated with disease in case−control studies (*38*) and affect susceptibility to and severity of meningococcal disease. In our study, we found that persons <5 years of age who had a familial history of this disease had a 4-fold greater probability of developing it.

An age <1 year and being infected with serogroup W were strongly associated with the likelihood of meningococcal disease. Other risk factors were similar to those found by analysis of all other serogroups.

Strengths of this study were including 91% of all cases that occurred in the study period, selecting 4 population controls/case-patient, and sampling that was independent of exposure conditions. These features ensured comparability and that the controls could show development of the disease because they resided in municipalities in which cases of meningococcal disease were still occurring.

One limitation of this study was that, because of the case−control design, information about exposure was collected retrospectively. As a result, there might have been memory bias, the probability of which increases when information about several factors is collected simultaneously, as was the case in this study. A considerable amount of time passed between occurrence of disease and when the study was conducted. Another limitation was that controls were questioned about a period 2 years before they were interviewed, However, the study was conducted 6 months after the last case included in the study had occurred. The period during which case-patients and controls were investigated was different, although it might have included a common time frame.

The question of vaccination status was also a problem because a vaccination campaign occurred once the outbreak was recognized. Therefore, controls had an opportunity to be vaccinated that case-patients did not. Because the likelihood of vaccination was different for case-patients and controls, we did not analyze this variable.

If one considers that prospective designs are ideal, it would be useful to conduct a new study of incident cases during an outbreak to further explore the causality of associations identified in this study. In addition, the protective role of vaccination against meningococcus, which has been demonstrated worldwide, should stimulate discussion about which groups should be targeted for vaccination. Although children 12 months of age have been vaccinated in Chile since 2014, a booster vaccination during adolescence might be advisable, as recommended by the World Health Organization (*15*). Therefore, analysis of age groups most affected by serogroup W should be performed to determine whether vaccination is affecting the target group or a shift to other groups has occurred at the present time. Vaccination can compensate for risk factors and protect against this disease. These findings indicate the need for health systems to move toward health equity by targeting actions to the most vulnerable groups.

Results of this study have contributed to understanding the epidemiology of the disease and identified determinants and risk factors that increase the probability of development of meningococcal disease in the population in Chile. Unfavorable living conditions, such as poverty, small spaces, overcrowding, and a previous clinical history, especially respiratory sequelae, are major risk factors. Positive familial history of meningococcal disease also seems to be a major factor associated with development of the disease, especially in children, a finding that should be addressed in future research. Most of these indicators point to a highly vulnerable social group that should be the target of intersectoral public policies, enabling them to improve their living conditions and health.
